# Cellular miR-24-3p inhibits vaccinia virus replication by targeting kinesin-like protein KIF21B

**DOI:** 10.1016/j.bsheal.2026.05.002

**Published:** 2026-05-12

**Authors:** Pengtao Jiao, Ying Ran, Dan Liu, Lingling Mei, Gang Pang, Shuang Liang, Yang Liu, Lei Sun, Zhaohui Wang, Ke Zhang

**Affiliations:** a Institute of Animal Science, Chinese Academy of Agricultural Sciences, Beijing 100193, China; b Shanghai Institute of Materia Medica, Chinese Academy of Sciences, Shanghai 201203, China; c University of Chinese Academy of Sciences, Beijing 100049, China; d State Key Laboratory of Bioactive Substance and Function of Natural Medicines, Institute of Materia Medica, Chinese Academy of Medical Sciences & Peking Union Medical College, Beijing 100050, China; e Institute of Infectious Diseases, Shenzhen Bay Laboratory, Shenzhen 518107, China; f Institute of Microbiology, Chinese Academy of Sciences, Beijing 100101, China; g Shanghai Institute of Immunity and Infection, Chinese Academy of Sciences, Shanghai 200031, China

**Keywords:** Vaccinia virus (VACV), Microribonucleic acids (miRNAs), miRNA-24-3p (miR-24-3p), KIF21B

## Abstract

Microribonucleic acids (miRNAs) play diverse roles in numerous biological processes. miRNA-24-3p (miR-24-3p) has been reported to play an important role in viral infection. However, little is known about the involvement of miR-24-3p in persistent vaccinia virus infection. In this study, we discovered that vaccinia virus Western Reserve (VACV-WR) infection suppressed miR-24-3p expression. Delivery of synthetic miR-24-3p mimics into cells reduced viral genome replication, protein levels, and viral titers in VACV-WR-infected cells. Target prediction analysis identified KIF21B as a host target of miR-24-3p, and KIF21B deficiency significantly decreased VACV-WR replication and infection, suggesting that KIF21B is an important host factor facilitating VACV replication. Finally, in a VACV-infected mouse model, miR-24-3p was delivered using lipid nanoparticles (LNP), resulting in attenuated weight loss, higher survival rates, and lower viral loads, confirming that miR-24-3p overexpression significantly restricts VACV replication. In summary, our study demonstrates that miR-24-3p targets the host KIF21B sequence to coordinate suppression of VACV replication, providing a potential therapeutic strategy for VACV treatment.

## Highlights

• **Scientific questions:** Ongoing viral transmission and the increasing threat of drug resistance highlight the pressing need for the development of new anti-monkeypox virus therapies. The roles of microribonucleic acids (miRNAs), which regulate gene expression at the post-transcriptional level, in poxvirus infection remain poorly understood and require further investigation.
• **Evidence before this study:** Increasing evidence has shown that miRNAs are key regulators of gene expression. A growing number of miRNAs have been implicated in various pathological conditions, including viral infections. Previous studies reported that miR-24-3p inhibits the replication of vesicular stomatitis virus (VSV), hepatitis C virus (HCV), H5N1 influenza A virus, and severe acute respiratory syndrome coronavirus 2 (SARS-CoV-2).
• **New findings:** miR-24-3p expression is significantly reduced during vaccinia virus (VACV) infection, whereas enforced overexpression markedly inhibits VACV replication, identifying miR-24-3p as a functional antiviral miRNA in poxvirus infection. KIF21B is identified as a promoter of VACV replication, uncovering a novel miRNA-host factor axis (miR-24-3p-KIF21B) that regulates VACV replication. Lipid nanoparticle-mediated delivery of miR-24-3p confers significant protection in a VACV-infected mouse model, providing *in vivo* evidence for miRNA-based therapy against poxvirus infection.
• **Significance of the study:** These findings broaden our knowledge of the interplay between miRNAs and VACV, fill a critical knowledge gap in poxvirus biology, and highlight miR-24-3p as a promising therapeutic candidate for the prevention and treatment of orthopoxvirus infections.

## 1 Introduction

Poxviruses are recognized as the largest viruses and exhibit an unparalleled level of structural complexity. Infection in humans and animals typically leads to localized or systemic suppurative skin lesions. Among poxviruses, eight genera are known to infect mammals: *Orthopoxvirus*, *Capripoxvirus*, *Avipoxvirus*, *Parapoxvirus*, *Suipoxvirus*, *Leporipoxvirus*, *Molluscipoxvirus*, and *Yatapoxvirus*. The genus *Orthopoxvirus* (OPXV) includes mpox virus (MPXV), cowpox virus (CPXV), vaccinia virus (VACV), variola virus (VARV), camelpox virus (CMPV), ectromelia virus (ECTV), and taterapox virus (TATV) [1], [2]. Of these, four are recognized as human pathogens: MPXV, VARV, VACV, and CPXV [3]. The recent multinational outbreak of mpox marks the largest and most widespread epidemic since the virus was first identified. International spread across multiple countries has been driven by human-to-human transmission, likely initiated by several imported cases in adults. The absence of a clearly defined epidemiological transmission chain constitutes a considerable public health concern. Currently, several targets at different stages of the MPXV life cycle have been identified as anti-viral treatment targets [4]. Cidofovir is a nucleoside analogue that targets viral deoxyribonucleic acid (DNA) polymerase. To reduce its nephrotoxicity, structural analogues have been developed, such as brincidofovir, which incorporates lipid conjugation technology to improve safety and bioavailability [5], [6], [7], [8]. Tecovirimat, which targets the MPXV VP37 protein to block viral egress and cell-to-cell spread, received emergency use authorization from the Food and Drug Administration for use in severe cases or in high-risk patients under expanded access [9], [10], [11], [12]. Ongoing viral transmission and the increasing threat of drug resistance highlight the pressing need for the development of new anti-MPXV therapies [13].

Small nucleic acids, including antisense oligonucleotides, small interfering ribonucleic acids (siRNAs), microRNAs (miRNAs), short hairpin RNAs (shRNAs), and aptamers, are key regulators of gene expression. Benefiting from their intrinsic specificity achieved through sequence-complementary base pairing or structural recognition, their programmability, and advances in efficient delivery systems, small nucleic acid-based therapeutics have increasingly been translated into clinical applications [14]. In recent years, increasing evidence has shown that miRNAs are key regulators of development, function, and homeostasis across diverse tissues and organisms [15], [16]. miRNAs are a class of small non-coding RNAs of approximately 18-25 nucleotides that control gene expression at the post-transcriptional level by targeting messenger RNAs (mRNAs) for decay or translational suppression [17]. Through sequence-specific base pairing, miRNAs can silence or fine-tune the expression of their target genes [18], [19], [20]. A growing number of miRNAs have been implicated in various pathological conditions-including cardiovascular diseases [21], [22], viral infections [23], and cancers-spurring increasing interest in their biological roles within specific cellular contexts. Although preclinical research on miRNA drugs has advanced considerably, no miRNA drugs have yet been approved [24]. Among miRNA-based therapies that have entered clinical trials, RG-101, which is an inhibitor of miR-122, advanced to Phase II for the treatment of hepatitis C virus (HCV), highlighting the potential for translating miRNA therapeutics into antiviral treatments [25], [26], [27]. Previous studies have shown that miRNA has broad-spectrum antiviral effects by regulating host gene expression, including primate foamy retrovirus [28], vesicular stomatitis virus (VSV) [29], human immunodeficiency virus (HIV) [30], [31], influenza A virus (IAV) [32], japanese encephalitis virus [33], pseudorabies virus [34], dengue virus [35], and infectious bursal disease virus [36].

miRNA-24-3p (miR-24-3p), a conserved miRNA belonging to the miR-24 family, participates in multiple cellular and physiological processes [37]. Previous studies reported that miR-24-3p reduces VSV infectivity *in vivo* [29]. Additional studies have shown that miR-24-3p also inhibits the replication of HCV, IAV, and severe acute respiratory syndrome coronavirus 2 (SARS-CoV-2) [38], [39], [40]. However, whether miRNAs can inhibit OPXV, particularly the MPXV, has not yet been reported. KIF21B, a motor protein belonging to the kinesin‑4 family, plays an important role in regulating microtubule dynamics [41]. However, its function in viral infection has not yet been established.

In this study, we demonstrate that the mammalian miR-24-3p inhibits VACV replication both *in vitro* and *in vivo*. Mechanistically, miR-24-3p targets the host gene KIF21B, which we identified as an important host factor that promotes VACV replication. miR-24-3p suppresses KIF21B expression, thereby attenuating viral replication. Our findings indicate that miR-24-3p restricts viral propagation in cell culture and reduces disease severity in mice, thereby underscoring its promise as a therapeutic strategy against VACV infections.

## 2 Materials and methods

### 2.1 Cells and viruses

The A549, BHK21, and 293T cells were cultured in Dulbecco’s modified Eagle medium (DMEM, Gibco, USA) supplemented with 10% fetal bovine serum (FBS, Gibco, USA) at 37 °C in 5% CO_2_. Vaccinia virus Western Reserve (VACV-WR) strain and the recombinant WR expressing green fluorescent protein (VACV-WR-GFP) are gifted from the Institute of Microbiology, Chinese Academy of Sciences, and propagated in BHK21 cells. Virus infections were performed in medium containing 2% fetal bovine serum (FBS). Viral titers in cell lysates, prepared by three rounds of freeze-thaw cycles to disrupt the cells, were determined by the standard plaque assay in BHK21 cells.

### 2.2 Reagents and antibodies

Lipofectamine 3000 (Cat # L3000015), Lipofectamine™ RNAiMAX (Cat # 13778150), and TRIzol (Cat # 15596018CN) reagents were purchased from Thermo Fisher Scientific. The anti-KIF21B rabbit polyclonal antibody (Cat # AC002) was purchased from Sangon Biotech. Anti-A35 and anti-C5 antibodies were generated by immunizing mice with purified recombinant proteins expressed in *Escherichia coli*. The glyceraldehyde-3-phosphate dehydrogenase (GAPDH) antibody (Cat # AC002) was purchased from ABclonal. Horseradish peroxidase (HRP)-conjugated anti-mouse secondary antibody (Cat # M21001L), HRP-conjugated anti-rabbit secondary antibody (Cat # M21002L) were obtained from Abmart.

### 2.3 Plasmids, miRNAs, and siRNA

Wild-type and mutant miR-24-3p target sequences in the KIF21B 3′-untranslated region (UTR) were cloned into the UTR luciferase reporter plasmid pmirGLO (Promega).

The miR-24-3p mimic and inhibitor were purchased from GenePharma (Shanghai, China). The sequence of the double-stranded miR-24-3p mimic was: UGGCUCAGUUCAGCAGGAACAG (sense) and GUUCCUGCUGAACUGAGCCAUU (antisense); and the sequence of the miR-24-3p inhibitor was CUGUUCCUGCUGAACUGAGCCA.

siRNAs targeting KIF21B were synthesized by GENEWIZ (Suzhou, China). The sequences were as follows: siKIF21B-1: GGAGCUGAUGGAGUAUAAGTT (sense) and CUUAUACUCCAUCAGCUCCTT (antisense); siKIF21B-2: GGACCUGAGUUCAAAGUCATT (sense) and UGACUUUGAACUCAGGUCCTT (antisense); and siKIF21B-3: GCUACGGACUAAGCUUCUATT (sense) and AUGCUCCAGUGACUUCUCCTT (antisense).

### 2.4 Cell viability assay

A549 cells seeded in 96-well plates were transfected with miRNA or siRNA, and the cell viability was measured at 48 h post-transfection using a colorimetric-based cell counting kit-8 (CCK-8) assay (Dojindo Molecular Technologies, Cat # CK04). 10 μL of CCK-8 reagent was added to each well, followed by incubation at 37 °C for 1 h. The absorbance at 450 nm was then measured using a microplate reader.

### 2.5 Dual-luciferase assay

For the dual-luciferase assay, 293T cells were seeded in 24-well plates and transfected with luciferase reporter vectors together with 50 nmol/L miR-24-3p mimic using Lipofectamine 3000. At 24 h post-transfection, Cellular extracts were collected and subjected to analysis using a Dual-Luciferase Assay Kit (Beyotime, Cat # RG027). Data are shown as relative luciferase activity (Firefly luciferase normalized to Renilla luciferase), with each treatment carried out in triplicate in three independent experiments.

### 2.6 RNA isolation and quantitative real-time polymerase chain reaction (PCR) analysis

Total cellular RNA was extracted using TRIzol reagent and reverse transcribed into complementary DNA (cDNA) using the TransScript® One-Step genomic DNA (gDNA) Removal and cDNA Synthesis SuperMix (TransGen Biotech, Cat # AT311-04). Quantitative real-time PCR was performed with PerfectStart® Universal Green quantitative PCR (qPCR) SuperMix (TransGen Biotech, Cat # AQ601-04-V2) on an Applied Biosystems 7500 Real-Time PCR System (Applied Biosystems, USA). For relative quantification, gene expression levels were normalized to GAPDH or U6. The specific primers used are in Table 1.

**Table 1:** Sequences of quantitative polymerase chain reaction primers.

Genes	Primer (forward)	Primer (reverse)
*A35R*	TTTGTGCCGCATACAGATCA	CCATCCATTGCCGTCTGATAT
*A27L*	ATGGACGGAACTCTTTTCCCC	TGCTTCGCGTTTAGCCTCTG
*L1R*	TTGGCAGCGTTGTTTATGTACT	GGTAGCAATAACCATCGGAGAA
*GAPDH*	GGGAGCCAAAAGGGTCATCA	AGTGATGGCATGGACTGTGG
*SYT7*	AGACCTCGTCAACTCCCTCAC	TGTAGCCGACACTGAACTGGA
*DIAPH1*	CAGTTGGGTGCAAACATTTGG	TCCGGCTATCGTAACTCCCAG
*SNX13*	GGGGTTTAGTGGTTACTCTCCT	CCTGGCTTCCCGTTTCATTTC
*TRIM11*	GAGAACGTGAACAGGAAGGAG	CCATCGGTGGCACTGTAGAA
*MAPK14*	GCATAATGGCCGAGCTGTTG	TCATGGCTTGGCATCCTGTT
*RC3H1*	CTCCCTGGTCATGTGACACC	CGCTGGTCCCTCATTCCTTT
*KIF21B*	CGACTCCTCTTTGTCGGAGG	ATGTAGGAGGTGGACACGGA
*EYA4*	GAAAGAGCTGCGGGAAAAGC	GCCACCCCACAACAAATAGC

### 2.7 Plaque assay

BHK21 cell monolayers (5×10^6^ cells at 90% confluence in a 12-well plate) were infected with serial dilutions of virus for 2 h at 37 °C. Following infection, the virus inoculum was removed and the cells were washed twice with phosphate-buffered saline (PBS). The cell monolayers were then overlaid with a microcrystalline cellulose medium composed of DMEM supplemented with 2% FBS, subsequently incubated at 37 °C until plaques became visible at 72 hour post infection (h.p.i.). Plaques were then counted, and viral titers were calculated accordingly.

### 2.8 Western blot

Cells were washed twice with ice-cold phosphate-buffered saline (PBS) and lysed in 2×sample buffer [125 mmol/L Tris-HCl, pH=6.8; 20% glycerol; 13% sodium dodecyl sulfate (SDS)] containing protease inhibitors (TransGen Biotech, Cat # DI111-02). Protein extracts were denatured at 95 °C for 5 min in SDS-polyacrylamide gel electrophoresis (PAGE) loading buffer. Equivalent amounts of protein were resolved by SDS-PAGE and then transferred onto polyvinylidene fluoride (PVDF) membranes (Millipore, Cat # 24937-79-9). The membranes were incubated with the specified primary antibodies and then with HRP-conjugated secondary antibodies. Protein signals were visualized using Chemiluminescent HRP Substrate (Tanon, Cat # 180-5001) and captured with the Tanon 5200 Multi Fully Automated Chemiluminescence/Fluorescence Image Analysis System. Band intensities were quantified using ImageJ software, with GAPDH serving as an internal control.

### 2.9 Immunofluorescence microscopy

A549 cells transfected with miRNA or siRNA were infected with VACV-GFP-WR at a multiplicity of infection (MOI) of 0.1 for 24 h. Cells were then fixed with 4% paraformaldehyde (Biosharp, Cat. # BL539A) for 10 min, and nuclei were stained with 4',6-diaminyl-2-phenylindole (DAPI, Invitrogen, Cat. #H3579) for 10 min at room temperature. Images were captured using an Olympus IX73 Inverted Fluorescence Microscope (Olympus IX73, Japan).

### 2.10 Fabrication and characterization of lipid nanoparticles

A lipid mixture was prepared by combining 13.3 μL of (6Z,9Z,28Z,31Z)-heptatriaconta-6,9,28,31-tetraen-19-yl 4-(dimethylamino) butanoate (DLin-MC3-DMA, 116.8 mmol/L, MedChemExpress, Cat # HY-112251 ), 24.6 μL of 1,2-distearoyl-sn-glycero-3-phosphocholine (DSPC, 12.7 mmol/L, Avanti Polar Lipids, Cat # 850365,), 46.4 μL of cholesterol (25.9 mmol/L, Sigma-Aldrich, Cat # C3045), 11.7 μL of 1,2-dimyristoyl-sn-glycero-3-methoxypolyethylene glycol (DMG-PEG2000, 4.0 mmol/L, Avanti Polar Lipids, Cat # 880151), with the volume adjusted to 18 μL using ethanol. All of the above solutions were prepared in ethanol. Separately, 8 μL of miRNA (1 μg/μL, GenePharma, China) was diluted in 46 μL of 0.1 mol/L citrate buffer (pH 4.4, Beijing solaibao technology, China) to form the aqueous phase. The RNA solution was rapidly added to the lipid mixture under vigorous pipetting for 20-30 s, followed by incubation at room temperature for 15 min. The dialysate was collected and adjusted to a final volume of 800 μL to obtain the lipid nanoparticles. The size of the nanoparticles was measured by dynamic light scattering (DLS, Malvern Instruments Ltd., UK), and surface morphology was observed by transmission electron microscopy (TEM, JEOL JEM-100CX-II, Japan).

### 2.11 Mouse experiments

Six-week-old female BALB/c mice (Vital River Laboratory, Beijing, China) were injected with PBS or a miRNA-lipid nanoparticles solution (19.8 ug/100 uL) *via* the tail vein. The first injection of each miRNA-lipid nanoparticles solution was designated as day 0, and booster injections were administered on days 3, 6, and 9 at the same dose (19.8 μg/100 μL). Two hours post-injection, they were infected intraperitoneally with 5×10^6^ plaque-forming unit (PFU) of VACV in an animal biosafety level 2 facility at the Institute of Biophysics, Chinese Academy of Sciences. Body weight and survival were monitored for 14 days post-challenge. On day 6 post-challenge, lung, spleen, and liver tissues were collected for analyses of viral titers, mRNA and protein expression levels, and histopathological alterations. Mice were euthanized with CO_2_ upon reaching the ethical endpoint of 30% body-weight loss.

### 2.12 Statistical analysis

Data are presented as mean ± standard deviation (SD). Statistical analyses were performed using GraphPad Prism 8.4.2. An unpaired two-tailed Student’s *t*-test, multiple *t*-tests, one-way analysis of variance (ANOVA) with Tukey’s post hoc test, or Log-rank (Mantel-Cox) test was used as appropriate. *P* value < 0.05 was considered significant. Statistical significance was defined as * *P* < 0.05, ** *P* < 0.01, and *** *P* < 0.001.

## 3 Results

### 3.1 miR-24-3p restricts VACV replication *in vitro*

**Fig. 1:**
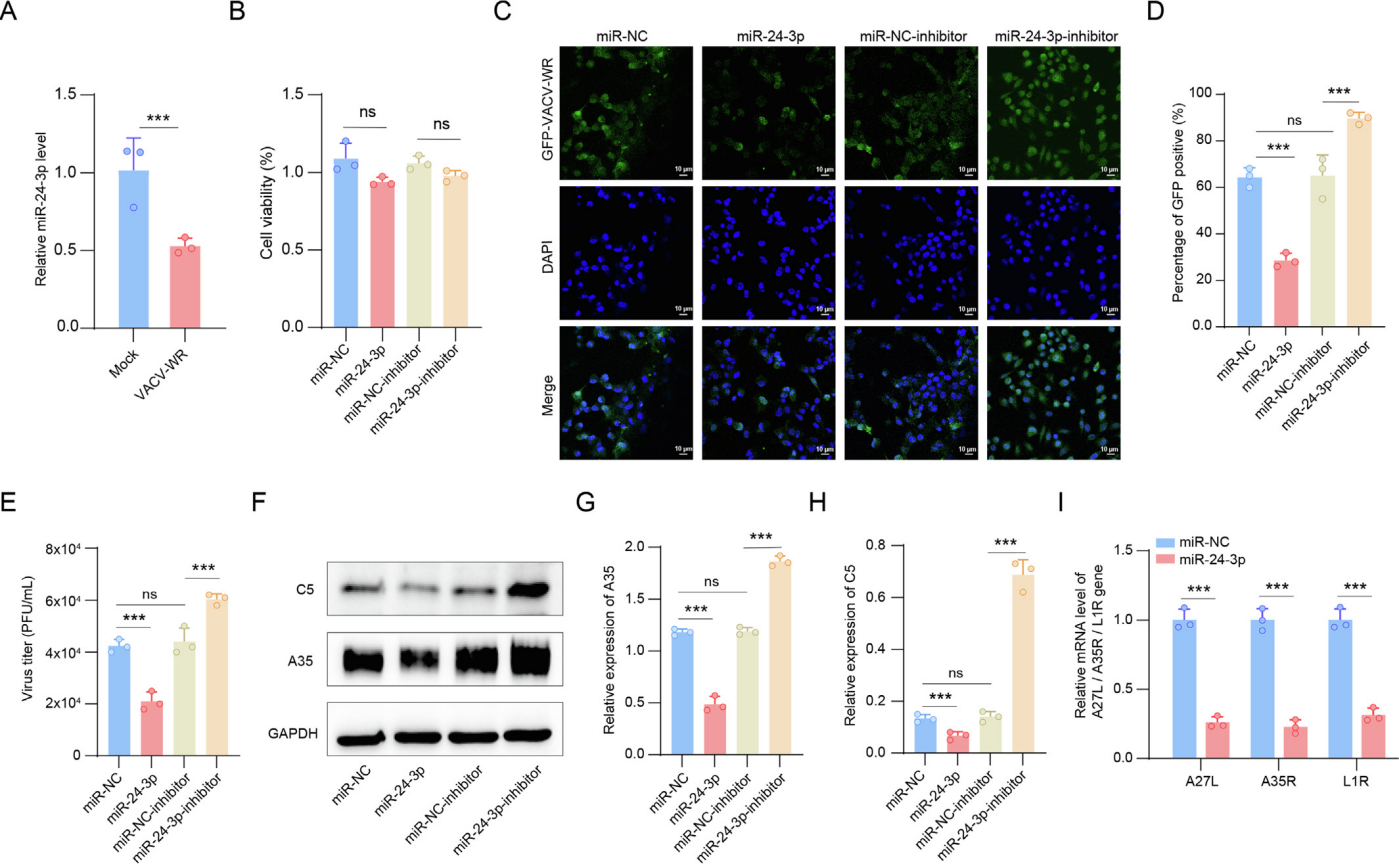
miR-24-3p restricts VACV replication *in vitro*. A) A549 cells were infected with VACV at an MOI of 1. Total RNA was extracted at 16 h post-infection, and miR-24-3p expression levels were measured by RT-qPCR. U6 small nuclear RNA was used as the normalization control. B) A549 cells were transfected with miR-24-3p mimics (40 nmol/L) or inhibitors (100 nmol/L). Following 48 h of transfection, the CCK-8 assay was used to evaluate cell viability. C–D) A549 cells were transfected with miR-24-3p mimics (40 nmol/L) or inhibitors (100 nmol/L) and infected with VACV-WR-GFP at an MOI of 0.1 for 24 h. VACV-WR-GFP expression was monitored using a fluorescence microscope (C); The intensity of GFP fluorescence was measured and quantified using ImageJ software (D). E–I) A549 cells were transfected with miR-24-3p mimics (40 nmol/L) or inhibitors (100 nmol/L) and infected with VACV-WR at an MOI of 0.1 for 24 h. Viruses in A549 cells were harvested and quantified by plaque assay on BHK21 cells (E); Cell lysates were collected, and VACV protein levels were analyzed by western blot using anti-C5 and anti-A35 antibodies (F); Band intensities for A35 (G) and C5 (H) were quantified separately using ImageJ software (G); Total RNA was harvested, and viral mRNA levels were measured by RT-qPCR, with GAPDH used as the normalization control (I). Data are shown as mean ± SD. Statistical significance was analyzed by a two-tailed Student’s *t*-test (A, I) or one-way ANOVA followed by Tukey’s multiple comparison test (B, D, E, G, H) (*, P < 0.05; ***, P < 0.001; ns, not significant). Abbreviations: MOI, multiplicity of infection; RT-qPCR, reverse transcription quantitative polymerase chain reaction; VACV, vaccinia virus; VACV-WR, vaccinia virus Western Reserve; RNA, ribonucleic acid; GFP, green fluorescent protein; GAPDH, glyceraldehyde-3-phosphate dehydrogenase; CCK-8, colorimetric-based cell counting kit-8; miR, micro-ribonucleic acids; miR-NC, miRNA control; DAPI, 4′,6-diamidino-2-phenylindole; SD, standard deviation; ANOVA, analysis of variance.

Previous studies have identified a series of miRNAs resistant to VSV infection. Among these, miR-24-3p stands out as one of the most robust responders, having been implicated in suppressing viral replication across multiple viral infections [29], [38], [39], [40]. The miR-24-3p level was examined following VACV-WR infection by qPCR. The results showed that VACV-WR infection significantly downregulated miR-24-3p expression compared with the mock group, suggesting that miR-24-3p may suppress VACV-WR replication (Fig. 1A). To determine the role of miR-24-3p in VACV replication, miRNA control (miR-NC), miR-24-3p mimics, miR-NC-inhibitor, or miR-24-3p inhibitor was transfected into A549 cells, followed by VACV-WR infection. First, the cytotoxicity of these miRNA modulation reagents was evaluated. Transfection with exogenous miRNA mimics or inhibitors did not affect the viability of A549 cells (Fig. 1B). Cells were then infected with VACV-WR-GFP, and infection efficiency was monitored by fluorescence microscopy (Fig. 1C). Quantification of GFP-positive cells revealed that miR-24-3p overexpression significantly reduced the number of GFP-positive cells compared with the miR-NC, whereas inhibition of miR-24-3p increased the proportion of GFP-positive cells (Fig. 1D). Viral titers in cells were determined by plaque assay, revealing that the miR-24-3p mimic significantly inhibited VACV-WR replication (Fig. 1E). Viral proteins expression in VACV-WR infected cells were detected by western blot using anti-C5, and anti-A35 antibodies (Fig. 1F). Quantification showed that miR-24-3p mimic treatment markedly decreased C5 and A35 protein levels, whereas the miR-24-3p inhibitor significantly increased their expression (Fig. 1G and 1H). The inhibitory effect of miR-24-3p on VACV genome replication was further confirmed by qPCR using primers targeting A27L, A35R, and L1R genes (Fig. 1I). Collectively, these results indicate that miR-24-3p exerts an inhibitory role in VACV infection.

### 3.2 miR-24-3p targets KIF21B and negatively regulates its expression

**Fig. 2:**
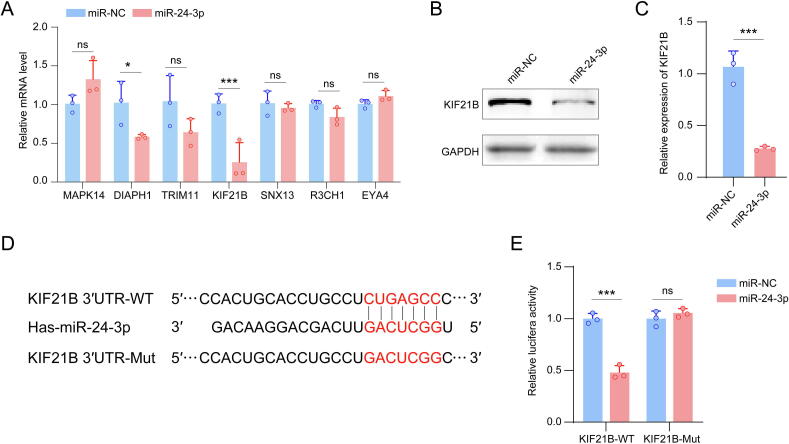
miR-24-3p targets kif21b and negatively regulates its expression. A) Following transfection of A549 cells with 40 nmol/L miR-24-3p mimics or negative control, RT-qPCR was used to measure the mRNA levels of the indicated potential target genes at 24 h post-transfection. GAPDH was used as the normalization control. B) A549 cells were transfected with 40 nmol/L miR-24-3p mimics or negative control for 48 h. Cell lysates were then collected, and KIF21B protein levels were analyzed by western blot using an anti-KIF21B antibody. C) Band intensities from (B) were quantified using ImageJ software. D) Sequence of the computationally predicted miR-24-3p binding site in the KIF21B 3′-UTR and the corresponding mutant sequence used in the experiments shown in (E). E) HEK293T cells were co-transfected with 50 nmol/L miR-24-3p mimic or negative control together with the reporter plasmid KIF21B-WT or KIF21B-mut. Cell lysates were harvested 24 h post-transfection for dual-luciferase assays. Data are shown as mean ± SD. Statistical significance was analyzed by a two-tailed Student’s *t*-test (A and C) or multiple *t*-tests (E) (*, *P* < 0.05; ***, *P* < 0.001; ns, not significant). Abbreviations: RT-qPCR, reverse transcription quantitative polymerase chain reaction; VACV, vaccinia virus; VACV-WR, vaccinia virus Western Reserve; mRNA, messenger ribonucleic acid; GAPDH, glyceraldehyde-3-phosphate dehydrogenase; UTR, untranslated region; miR, microribonucleic acids; miR-NC, miRNA control; SD, standard deviation.

Growing evidence suggests that miRNAs influence cellular processes by interacting with the 3′ untranslated regions of their target mRNAs to regulate gene expression [42], [43]. Consequently, potential target genes of miR-24-3p were identified using the online miRNA database, TargetScan (www.targetscan.org). Bioinformatics analysis revealed that *SYT7, SNX13, EYA4, MAPK14, R3CH1, KIF21B, TRIM11, and DIAPH1* were potential target genes of miR-24-3p. To validate these targets, A549 cells were transfected with either miR-NC or miR-24-3p mimics for 48 hours, and the mRNA levels of the candidate genes were analyzed by qPCR. The results showed that DIAPH1, and KIF21B expression levels were downregulated by miR-24-3p, with KIF21B exhibiting the most pronounced reduction (Fig. 2A). Consistently, western blot analysis demonstrated that KIF21B protein levels were reduced in A549 cells upon miR-24-3p overexpression (Fig. 2B). Quantification revealed that KIF21B protein levels in miR-24-3p-transfected cells were reduced to approximately 25% of those in miR-NC transfected cells (Fig. 2C). Next, we employed the bioinformatics analysis tools StarBase (http://starbase.sysu.edu.cn/starbase2/) and TargetScan (http://www.targetscan.org) to predict the putative KIF21B binding sites within miR-24-3p. The analysis revealed a conserved miR-24-3p binding site located in the 3′-UTR of KIF21B. Accordingly, a mutant KIF21B 3′UTR construct was designed to disrupt the predicted miR-24-3p binding site (Fig. 2D). Further investigation of the interaction between miR-24-3p and KIF21B was performed using a luciferase reporter assay, in which 293T cells were co-transfected with either miR-NC or miR-24-3p mimics together with luciferase constructs encoding the wild-type or mutant KIF21B 3′-UTR. The results showed that miR-24-3p overexpression significantly reduced the luciferase activity of KIF21B-WT, whereas no appreciable change was observed for KIF21B-Mut (Fig. 2E). These results indicate that KIF21B is a direct downstream target of miR-24-3p and suggest that KIF21B gene may function as a host factor that facilitates VACV replication.

### 3.3 KIF21B functions as a critical host factor facilitating VACV replication

**Fig. 3:**
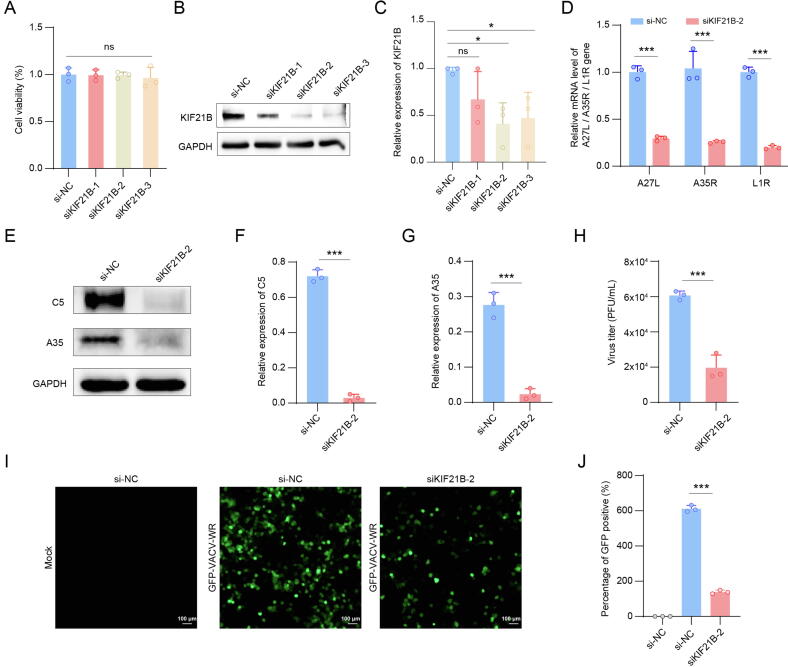
KIF21B functions as a critical host factor facilitating VACV replication. A) Cell viability was determined 48 h after transfection with the indicated siRNA using the CellTiter-Blue Cell Viability Assay. B) After 48 h of transfection, cell lysates were harvested, and KIF21B protein levels were determined by western blot using an anti-KIF21B antibody. C) Band intensities from (B) were determined by ImageJ software. D–H) A549 cells were transfected with siRNA designed to target KIF21B (siKIF21B-2) or a negative control, followed by infecting with VACV-WR at an MOI of 0.1 for 24 h. Total RNA was harvested, and viral A27L, A35R, and L1R mRNA levels were determined by RT-qPCR, with GAPDH used as the normalization control (D); Cell lysates were collected, and VACV C5 and A35 protein levels were analyzed by western blot using the corresponding antibodies (E); Band intensities for C5 (F) and A35 (G) were quantified separately using ImageJ software. Viruses were harvested and quantified by plaque assay on BHK21 cells (H). I–J) A549 cells were transfected with siRNA designed to target KIF21B (siKIF21B-2) or a negative control, followed by infecting with VACV-WR-GFP at an MOI of 0.1 for 24 h. VACV-WR-GFP expression was monitored using a fluorescence microscope (I). GFP-positive cells were scored using ImageJ software (J). Data are shown as mean ± SD. Statistical significance was analyzed by a two-tailed Student’s *t*-test (D, F, G, H, and J) or one-way ANOVA followed by Tukey’s multiple comparison test (A and C) (*, *P* < 0.05; ***, *P* < 0.001; ns, not significant). Abbreviations: MOI, multiplicity of infection; RT-qPCR, reverse transcription quantitative polymerase chain reaction; VACV, vaccinia virus; VACV-WR, vaccinia virus Western Reserve; GFP, green fluorescent protein; RNA, ribonucleic acid; siRNA, small interfering RNA; mRNA, messenger RNA; GAPDH, glyceraldehyde-3-phosphate dehydrogenase; NC, negative control; SD, standard deviation; ANOVA, analysis of variance.

To investigate the role of KIF21B in VACV-WR replication, three siRNAs that specifically target the KIF21B gene were designed. Cell viability experiments showed that KIF21B-specific siRNAs did not affect the viability of A549 cells (Fig. 3A). The knockdown efficiency of these siRNAs was evaluated by transfecting them into A549 cells, followed by western blot analysis using an anti-KIF21B antibody (Fig. 3B). Quantification showed that all siRNAs reduced KIF21B expression, with siKIF21B-2 exhibiting the most significant knockdown efficiency (Fig. 3C). siKIF21B-2 was then used to examine whether KIF21B silencing in A549 cells affects VACV-WR replication. Viral genome replication was assessed by qPCR using primers targeting A27L, A35R, and L1R mRNAs. The results demonstrated that KIF21B knockdown effectively reduced the expression of these viral mRNAs (Fig. 3D). Viral protein expression was further evaluated by western blot using antibodies against C5 and A35 (Fig. 3E). Quantification analysis revealed that reduced KIF21B protein levels significantly decreased C5 and A35 protein abundance (Fig. 3F, 3G). Consistently, downregulation of VACV-WR gene expression led to a reduction in viral titer (Fig. 3H). We additionally visualized the effects of KIF21B knockdown on GFP-VACV-WR infection using immunofluorescence confocal microscopy (Fig. 3I). Quantification of GFP-positive cells indicated that KIF21B silencing significantly reduced the number of infected cells compared with the siRNA control (Fig. 3J). Together, these data suggest that KIF21B is an important host factor that promotes VACV replication.

### 3.4 miR-24-3p restricts VACV replication and pathogenesis *in vivo*

**Fig. 4:**
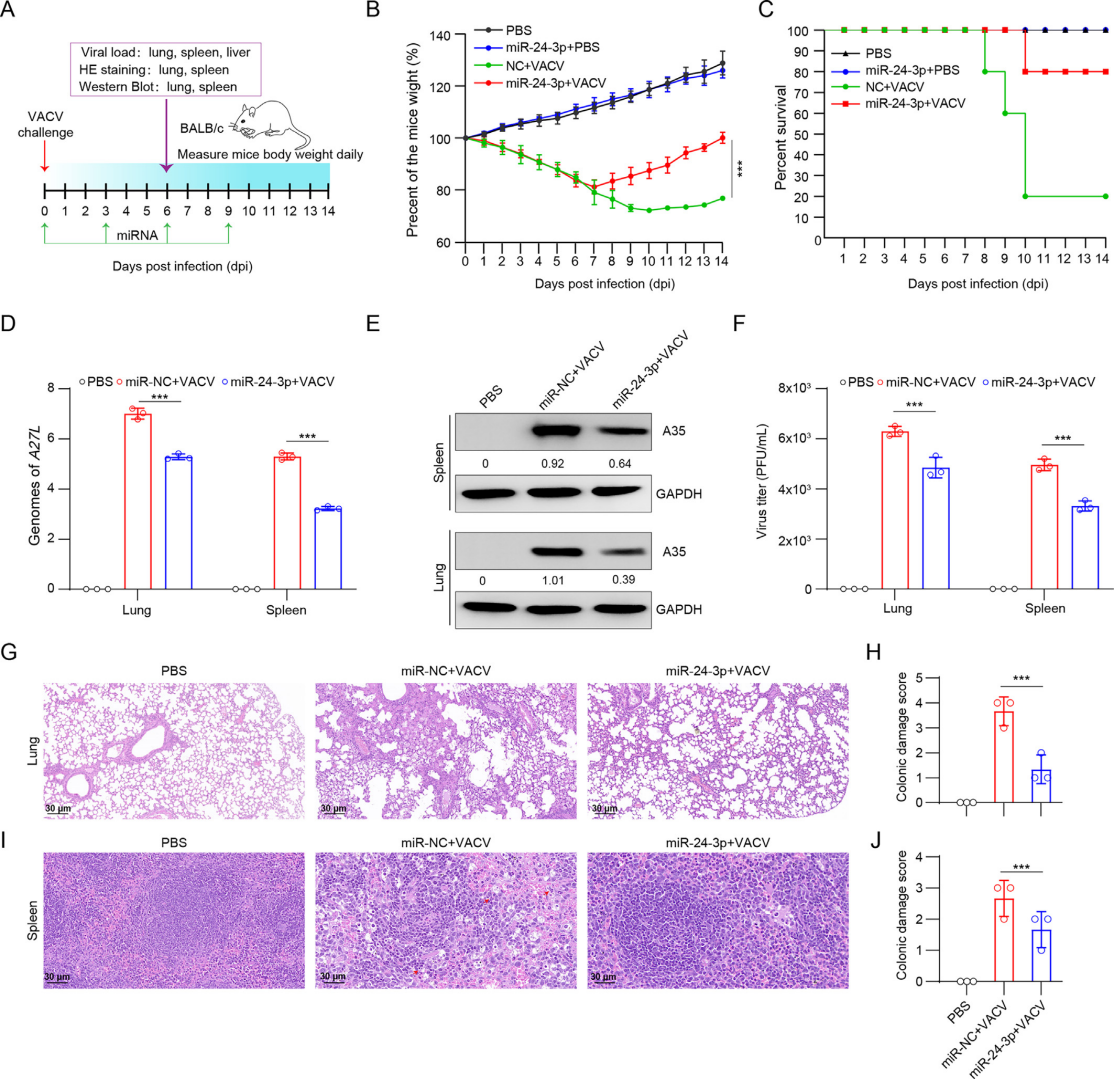
miR-24-3p restricts VACV-WR replication and pathogenesis *in vivo*. A) Schematic diagram of the mouse experiment used to evaluate the anti-VACV activity of miR-24-3p *in vivo*. Briefly, groups of mice were administered intravenous injections of PBS, miR-NC lipid nanoparticals, or miR-24-3p lipid nanoparticals, followed by intraperitoneal infection with VACV-WR (5 × 10^6^ PFU). Body weight changes and clinical signs were monitored for 14 days (n = 5). On day 6 post-infection, mice were euthanized, and the lungs, spleen, and liver were collected for further analysis (n = 3). B–C) Body weight changes (B) and survival rate (C) of mice after virus infection are shown. D) VACV A27L mRNA levels in the lungs and spleens of infected mice were quantified by RT-qPCR on day 6 post-infection, using GAPDH as an internal control. E) Immunoblot analysis of VACV-WR A35R protein level in the lungs and spleens of virus-infected mice. F) Infectious viral titers in lungs and spleens of virus-infected mice were determined by plaque assay. G) HE staining image (G) and colonic damage score (I) of the lungs and spleens of virus-infected mice. The severity of pathological manifestations was scored from 0 to 4, with 0 for minimal damage, 1 for mild damage, 2 for moderate damage, 3 for severe damage, and 4 for maximal damage. The scale bar was 30 µm. Data are shown as mean ± SD. Statistical significance was analyzed by one-way ANOVA followed by Tukey's multiple comparison test (D, F, H, and J); two-way repeated measures ANOVA (B); and Kaplan-Meier survival analysis followed by Log-rank (Mantel-Cox) test (C) (***, *P* < 0.001). Abbreviations: RT-qPCR, reverse transcription quantitative polymerase chain reaction; VACV, vaccinia virus; VACV-WR, vaccinia virus Western Reserve; RNA, ribonucleic acid; mRNA, messenger RNA; glyceraldehyde-3-phosphate dehydrogenase; NC, negative control; PBS, phosphate-buffered saline; PFU, plaque-forming unit; SD, standard deviation; HE staining, Hematoxylin and Eosin staining; ANOVA, analysis of variance.

To assess the impact of miR-24-3p on VACV-WR infection *in vivo*, miR-24-3p or a negative control (NC) was administered to BALB/c mice via tail vein injection, with PBS used as the control (Fig. 4A). Then, the effect of miR-24-3p on viral pathogenicity in the mice was examined. BALB/c mice were intraperitoneally infected with VACV-WR, and their body weight and survival were monitored daily. The results showed that the mice treated with miR-24-3p exhibited less body-weight loss than the mice treated with NC (Fig. 4B). Additionally, the mortality rate of the miR-24-3p-treated mice was lower than that of the mice treated with NC (Fig. 4C). To exclude the effect of miR-24-3p itself, the body weight and survival of mice injected with miR-24-3p in the absence of VACV challenge were also monitored. The results showed that the miR-24-3p-LNP complex did not affect body weight or survival (Fig. 4B, 4C). Viral gene expression in the lungs and spleens was assessed using primers targeting the A27L gene and an antibody against A35. The results showed that the DNA levels of A27L gene in the lungs and spleens of the miR-24-3p-treated mice were significantly lower than those in NC-treated mice at 6 days post infection (dpi) (Fig. 4D). Western blot analyses further revealed reduced A35R protein expression in the lungs and spleens of the miR-24-3p-treated group (Fig. 4E). Correspondingly, viral titers in both organs were decreased in mice treated with miR-24-3p (Fig. 4F). Histological examination of the lungs and spleens revealed that miR-24-3p-treated mice exhibited reduced alveolar damage and interstitial inflammatory cell infiltration compared with NC-treated mice at 6 dpi (Fig. 4G, 4I). The lung and spleen histopathological scores were significantly lower in miR-24-3p-treated mice compared with control mice (Fig. 4H, 4J). Taken together, these results suggest that miR-24-3p effectively reduces VACV-WR replication and pathogenesis *in vivo*.

## 4 Discussion

Therapeutic miRNAs represent a promising strategy to selectively modulate transcripts essential for viral replication [44]. Eukaryotic miRNAs play a key role in controlling a wide range of cellular physiological processes, including viral infections. Previous studies have demonstrated that miRNAs function as either proviral sensitizers or antiviral restrictors during viral replication, depending on the viral and cellular context [45], [46]. miRNAs that suppress viral replication have been identified in multiple viral infections, including IAV, HIV, HCV, Kaposi's sarcoma-associated herpesvirus (KSHV), respiratory syncytial virus (RSV), SARS-CoV-2, human cytomegalovirus (HCMV), and VSV, and represent potential therapeutic candidates for antiviral interventions [47], [48], [49], [50], [51], [52], [53], [54], [55], [56]. It has been reported that VACV infection regulates the expression of cellular miRNAs, indicating that miRNAs play important roles during VACV replication [57], [58]. However, the cellular miRNAs that inhibit poxvirus infection remain to be fully investigated. In this study, we confirmed that miR-24-3p acted as a potent restrictive factor against VACV-WR replication, which uncovered a novel regulatory axis distinct to poxvirus biology.

miRNAs suppress viral replication through multiple mechanisms. During viral replication, cellular proteins are involved at all stages of the viral life cycle. Targeting host factors required for the viral life cycle is one of the most important antiviral mechanisms [47]. miR-182 directly targets CLDN1, a tight-junction protein that functions as an essential entry co-receptor for HCV, thereby preventing viral entry [54]. miR-17 and miR-20, members of the miR-17-92 cluster, inhibit HIV transcription by targeting 3′-UTR of p300/CBP-associated factor (PCAF) mRNA[48]. miR-218-5p suppresses angiotensin-converting enzyme 2 (ACE2) expression by directly targeting ACE2 mRNA, thereby preventing SARS-CoV-2 entry [53]. Furin, neuropilin (NRP)1, NRP2, and sterol regulatory element-binding protein 2 (SREBP2) have been identified as entry factors for SARS-CoV-2, and miR-24-3p inhibits SARS-CoV-2 infection by downregulating the expression of these factors [38]. miR-24-3p also negatively regulates the p38 mitogen activated protein kinase (MAPK) signaling pathway by binding to 3′-UTR of MAPK14, resulting in the replication deficiency during IAV, RSV infection [52]. In this study, We identified the kinesin KIF21B as a direct target of miR-24-3p, with mimic transfection reducing both KIF21B mRNA and protein levels, confirmed by a luciferase reporter assay. Functionally, silencing KIF21B replicated the antiviral effect of miR-24-3p overexpression, effectively reducing viral DNA, protein expression, and titers, thus establishing KIF21B as a crucial pro-viral host factor. KIF21B is a microtubule-based motor protein that functions as a host factor involved in cytoskeletal dynamics and intracellular trafficking [59]. It has been reported that VACV hijacks host cytoskeletal systems to facilitate its intracellular transport, replication, and cell-to-cell spread [60]. Our findings suggest that this process also requires the involvement of additional host factors, such as KIF21B, providing new insights into the mechanisms underlying VACV infection.

Finally, in a BALB/c mouse model, administration of miR-24-3p alleviated VACV-WR pathogenicity, resulting in reduced weight loss, higher survival rates, lower viral loads and protein expression in the lungs and spleen, and significantly diminished lung tissue damage. In conclusion, our findings delineate a novel host defense mechanism wherein host-derived miR-24-3p restricts VACV replication by targeting the essential host factor KIF21B, both *in vitro* and *in vivo*, highlighting this axis as a potential therapeutic target. Given the ongoing threat of orthopoxvirus outbreaks and the limited arsenal of direct-acting antivirals, host-targeted strategies like miR-24-3p have distinct advantages in avoiding viral resistance and potentially covering multiple virus species.

miRNA-based therapeutics can be broadly classified into miRNA mimics, miRNA inhibitors (including antagomiRs and LNA-modified anti-miRs). Advances in delivery systems have substantially accelerated the development of miRNA-based therapeutics over the past decade[61]. In this study, miR-24-3p was delivered using lipid-based vectors, which may preferentially accumulate in the liver. Optimization of the delivery system could improve the therapeutic efficacy of miR-24-3p against VACV infection in future studies. For clinical translation, tissue-specific delivery and safety profiling will be critical steps to advance miR-24-3p toward in-human applications against poxvirus infections.

Evidence suggests that miRNAs from both the virus and host can regulate viral activity indirectly through diverse mechanisms, either enhancing or suppressing replication and influencing cell viability [62]. We have discovered that the high expression of the multifunctional miR-24-3p correlates with persistent infection, which is crucial for elucidating the pathogenesis of orthopoxviruses. In summary, our data indicate that miR-24-3p upregulation in cells persistently infected with VACV not only regulates the KIF21B gene involved in cellular signalling pathways but also suppresses viral replication, providing a potential therapeutic strategy for VACV treatment.

## Ethics statement

This study was approved and conducted strictly following the recommendations stated in the Guidelines for the Care and Use of Laboratory Animals issued by the Ethics Committee of the Institute of Biophysics, Chinese Academy of Sciences (ABSL-2-2024056).

## Acknowledgements

This work was funded by National Natural Science Foundation of China (82241078, 82372224, 82341052, 82473874, 82241082).

## Conflict of interest statement

The authors declare that there are no conflicts of interest.

## Author contributions

**Pengtao Jiao:** Methodology, Investigation, Data curation. **Ying Ran:** Writing – review & editing, Writing – original draft, Validation, Methodology, Investigation, Formal analysis, Data curation. **Dan Liu:** Methodology, Investigation, Data curation. **Lingling Mei:** Methodology, Investigation, Data curation. **Gang Pang:** Methodology, Investigation, Data curation. **Shuang Liang:** Methodology, Investigation, Data curation. **Yang Liu:** Validation, Funding acquisition, Formal analysis, Data curation. **Lei Sun:** Validation, Methodology, Formal analysis, Data curation. **Zhaohui Wang:** Methodology, Investigation, Funding acquisition. **Ke Zhang:** Writing – review & editing, Writing – original draft, Supervision, Investigation, Funding acquisition, Conceptualization.
